# Lineage replacement and evolution captured by 3 years of the United Kingdom Coronavirus (COVID-19) Infection Survey

**DOI:** 10.1098/rspb.2023.1284

**Published:** 2023-10-18

**Authors:** Katrina A. Lythgoe, Tanya Golubchik, Matthew Hall, Thomas House, Roberto Cahuantzi, George MacIntyre-Cockett, Helen Fryer, Laura Thomson, Anel Nurtay, Mahan Ghafani, David Buck, Angie Green, Amy Trebes, Paolo Piazza, Lorne J. Lonie, Ruth Studley, Emma Rourke, Darren Smith, Matthew Bashton, Andrew Nelson, Matthew Crown, Clare McCann, Gregory R. Young, Rui Andre Nunes dos Santos, Zack Richards, Adnan Tariq, Wellcome Sanger Institute COVID-19 Surveillance Team, COVID-19 Infection Survey Group, The COVID-19 Genomics UK (COG-UK) Consortium, Christophe Fraser, Ian Diamond, Jeff Barrett, Ann Sarah Walker, David Bonsall

**Affiliations:** ^1^ Big Data Institute, Nuffield Department of Medicine, University of Oxford, Old Road Campus, Oxford OX3 7LF, UK; ^2^ Department of Biology, University of Oxford, Oxford OX1 3SZ, UK; ^3^ Department of Mathematics, University of Manchester, Manchester M13 9PL, UK; ^4^ Wellcome Centre for Human Genetics, Nuffield Department of Medicine, NIHR Biomedical Research Centre, University of Oxford, Old Road Campus, Oxford OX3 7BN, UK; ^5^ Office for National Statistics, Newport, UK; ^6^ The Hub for Biotechnology in the Built Environment, Department of Applied Sciences, Faculty of Health and Life Sciences, Nothumbria University, Newcastle upon Tyne NE1 8ST, UK; ^7^ Department of Applied Sciences, Faculty of Health and Life Sciences, Northumbria University, Newcastle upon Tyne NE1 8ST, UK; ^8^ Wellcome Sanger Institute, Cambridge CB10 1SA, UK; ^9^ Nuffield Department of Medicine, University of Oxford, Oxford, UK; ^10^ The National Institute for Health Research Health Protection Research Unit in Healthcare Associated Infections and Antimicrobial Resistance, University of Oxford, Oxford, UK; ^11^ The National Institute for Health Research Oxford Biomedical Research Centre, University of Oxford, Oxford, UK; ^12^ Pandemic Sciences Institute, University of Oxford, Oxford, UK; ^13^ MRC Clinical Trials Unit at UCL, UCL, London, UK; ^14^ Oxford University Hospitals NHS Foundation Trust, John Radcliffe Hospital, Headington, Oxford OX3 9DU, UK; ^15^ Sydney Infectious Diseases Institute (Sydney ID), School of Medical Sciences, Faculty of Medicine and Health, University of Sydney, Sydney, Australia

**Keywords:** SARS-CoV-2, United Kingdom, Coronavirus (COVID-19) Infection Survey, evolution

## Abstract

The Office for National Statistics Coronavirus (COVID-19) Infection Survey (ONS-CIS) is the largest surveillance study of SARS-CoV-2 positivity in the community, and collected data on the United Kingdom (UK) epidemic from April 2020 until March 2023 before being paused. Here, we report on the epidemiological and evolutionary dynamics of SARS-CoV-2 determined by analysing the sequenced samples collected by the ONS-CIS during this period. We observed a series of sweeps or partial sweeps, with each sweeping lineage having a distinct growth advantage compared to their predecessors, although this was also accompanied by a gradual fall in average viral burdens from June 2021 to March 2023. The sweeps also generated an alternating pattern in which most samples had either S-gene target failure (SGTF) or non-SGTF over time. Evolution was characterized by steadily increasing divergence and diversity within lineages, but with step increases in divergence associated with each sweeping major lineage. This led to a faster overall rate of evolution when measured at the between-lineage level compared to within lineages, and fluctuating levels of diversity. These observations highlight the value of viral sequencing integrated into community surveillance studies to monitor the viral epidemiology and evolution of SARS-CoV-2, and potentially other pathogens.

## 1. Introduction

A crucial component of the global response to COVID-19 has been the identification, tracking and characterization of new SARS-CoV-2 lineages. As well as enabling researchers to identify patterns of spread, variants can be identified that might pose a particular risk. For instance, they may be able to transmit more easily, or evade immune responses. Prominent examples include the variants of concern (VOCs) Alpha, Beta, Gamma, Delta and Omicron [1,2], and individual mutations such as E484K, an immune escape mutation in the Spike protein [3,4]. As of April 2023, around 3 million SARS-CoV-2 sequences had been generated in the United Kingdom (UK) via the COVID-19 Genomics UK (COG-UK) Consortium [5] and the four UK Public Health Agencies, with this substantial surveillance effort generating a snapshot of the leading edge of infection across the UK.

Estimating the prevalence of SARS-CoV-2 lineages and/or mutations can, however, be subject to biases as a consequence of the sampling regime [6–11]. Sampling has been heavily focussed on symptomatic infections, even though a high proportion of infections are asymptomatic or may not reach the criteria for testing [12]. For example, in the early phase of the UK epidemic most testing was conducted among hospitalized patients with severe disease, with a later focus on symptomatic individuals. Where testing of asymptomatic individuals has been conducted, it has often been in the context of specific settings, such as returning travellers, schools, or as part of surge testing in geographical areas where VOCs have been identified [13]. Large-scale community surveillance studies, such as the Office for National Statistics Coronavirus (COVID-19) Infection Survey (ONS-CIS) [8] and the Real-time Assessment of Community Transmission (REACT) [14], are thus valuable since sampling is not subject to these biases, they consist of a random, potentially more representative sample of the population, and, crucially, identify both symptomatic and asymptomatic infections. Moreover, community-based surveillance studies are not reliant on sequencing samples collected as part of national RT-PCR testing programmes and may therefore become increasingly important as countries seek to enhance surveillance capabilities for SARS-CoV-2 and other pathogens.

The ONS-CIS is a UK household-based surveillance study, with households approached from address lists to ensure as representative a sample of the population as possible [8,15]. Sampling was undertaken continuously throughout the three years of the survey, with most participants assessed weekly for the first month after their enrolment and approximately monthly thereafter. This differs from REACT in which sampling was concentrated within multiple rounds, with each round lasting approximately 2–3 weeks, and with different individuals approached each round [14]. Over the course of the ONS-CIS, 11 264 965 swabs excluding voids were taken, of which 205 542 (1.8%) were positive for SARS-CoV-2 using RT-PCR (electronic supplementary material, figure S1).

Here, we present an analysis of the over 125 000 good quality consensus sequences from RT-PCR positive samples collected over the first three years of the ONS-CIS with the aim of reconstructing the key epidemiological and evolutionary features of the UK epidemic. These data captured the sequential sweeps and partial sweeps of the B.1.177, B.1.1.7/Alpha, B.1.617.2/Delta, and Omicon (BA.1, BA.2, BA.4, BA.5) lineages, the BA.2 recombinant lineage XBB, and BA.2.75 (a sublineage of BA.2) and BQ.1 (a sublineage of BA.5). Hereafter, we refer to these as major lineages, and for each, we calculated the growth rate advantage using a novel method based on Gaussian processes. This method has the benefit of providing smooth estimates for prevalence and growth rates at both the very low and sometimes zero case counts observed for some variants when they first emerge, and the very high counts once they are established. We also captured the curious alternation of sweeping lineages that exhibit RT-PCR S-gene target failure (SGTF) caused by the Spike DH69/V70 deletion, with those without the deletion, a pattern also observed in other countries such as South Africa [16].

Finally, we determined how these sweeps impacted measures of the genetic diversity and divergence of the virus, both at the within-lineage and between-lineage levels. Using only samples collected as part of the ONS-CIS, we observed a consistent pattern of low within-lineage diversity on first emergence, followed by a steady increase. To capture measures of divergence in a computationally efficient way, we downsampled sequences using weighted random sampling, enabling us to generate a phylogeny in which the major lineages, and VOCs which were sampled only rarely, were as evenly distributed through time as possible. Using linear regression we compared the overall rate of divergence of all downsampled sequences with those of the major lineages, with consistently much lower rates of evolution within than between lineages.

Although sequences from the ONS-CIS represented about 4% of the total number of SARS-CoV-2 sequences obtained in the UK during this period, and at times much <1%, we were able to reconstruct the key epidemiological and evolutionary aspects of the epidemic. Our observations highlight that representative sampling can capture key aspects of epidemics, and the important role that community-based genomic surveillance studies can have in the monitoring of infectious disease. Although the ONS-CIS is based in the UK, in which sequencing effort has been unprecedented, this is of particular importance in settings where routine testing is likely to be scaled back, and for countries exploring the best strategies for tracking SARS-CoV-2 as well as other respiratory pathogens in the future [1,17,18]

## 2. Results

### (a) Sequential replacement of lineages in the UK

Throughout the ONS-CIS, which was launched in April 2020, a selection of the samples positive by RT-PCR have been sequenced (electronic supplementary material, figure S1), and from December 2020 onwards the aspiration was to sequence all samples with Ct ≤ 30. Here we report on the sequenced samples with over 50% genome coverage collected between 26 April 2020 and 13 March 2023. The Pango lineage [19] for all the samples was determined using Pangolin [20], and from 7 December 2020 onwards we provided publicly available weekly reports, giving the breakdown of sequenced samples (with >50% genome coverage) by lineage [21].

Because ONS-CIS households are broadly representative of the UK population through the sampling design, and participants within these households are sampled regardless of symptoms or other factors, the proportion of RT-PCR positive samples in the survey is assumed to broadly reflect the proportion of infected individuals in the UK population as a whole. Participant characteristics can be found in [22], electronic supplementary material, tables S4–S6.

**Figure 1.**
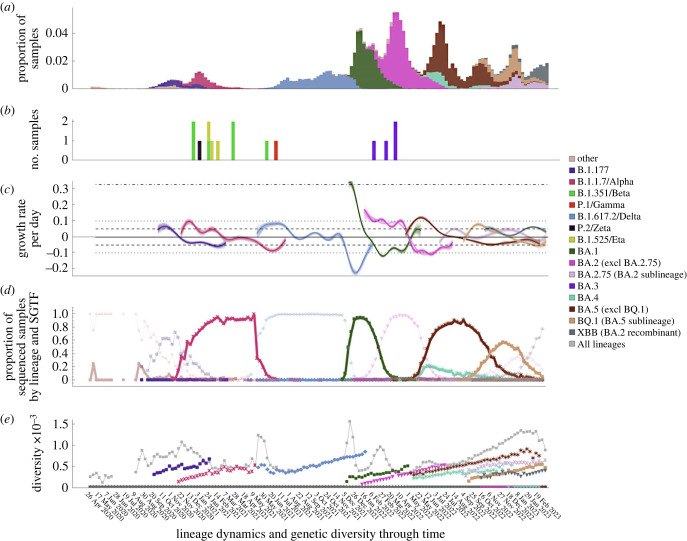
Lineage dynamics and genetic diversity through time. (*a*) Proportion of swabs taken as part of the ONS-CIS that were positive with Ct ≤ 30 (electronic supplementary material, figure S1), with bars coloured by the proportion of sequenced samples belonging to each major lineage. (*b*) Number of variant of concern (VOC) sequenced samples from rarer lineages (i.e. not classified as a major lineage). (*c*) Per day growth rate advantage of each of the major lineages compared to all other contemporary samples. BA.4 and BA.5 were considered together due to their concordant trajectories, and uncertainty is represented by 200 data bootstraps. The horizontal lines represent how long it would take the VOC prevalence to double (14 days, dashed; 7 days dotted; 2 days dot-dash). (*d*) Proportion of sequenced samples with both the given lineage and S-gene target failure (SGTF) pattern. The bold markers represent the proportion of sequenced samples that were of both the indicated lineage and had S-gene target failure (SGTF) during RT-PCR testing. The pale markers indicate those samples that were non-SGTF. (*e*) Genetic diversity among all samples and among samples of the same major lineages. Lineage designations include all sub-lineages except where indicated, and all samples were grouped by the week in which they were collected, with the date giving the first day of the collection week (every third week labelled for clarity).

The proportion of samples that were RT-PCR positive waxed and waned during the UK epidemic (figure 1*a*; electronic supplementary material, S1). There was a small 2020 autumn peak dominated by B.1.177 and its sub-lineages, followed by a decline in cases due to the second national lockdown which lasted from 5 November to 2 December 2020. Subsequent to this, the number of positives started to rise again. This rise is attributed to a relaxation of restrictions during the Christmas period and corresponded to a rapid rise in the number of B.1.1.7/Alpha infections. After the commencement of a further lockdown in England, Scotland and Northern Ireland in early January 2021, cases declined again, before another rapid increase in the number of sequenced samples that were dominated by B.1.617.2/Delta, with this increase corresponding to a phased reopening on 19 May and 20 June 2021. Three larger peaks in infections were observed between December 2021 and August 2022, each associated with the sequential emergence of the major Omicron lineages (BA.1, BA.2 and BA.4 and BA.5). Two subsequent smaller waves were not associated with any one lineage, but were dominated by sublineages of BA.2 and BA.5, before a final wave in which XBB, a recombinant BA.2 lineage, gained the advantage. Notably these three final waves all had higher peaks than the earlier B.1.1.77, B.1.1.7/Alpha, and B.1.617.2/Delta waves.

### (b) Sweeping lineages each had a substantial growth rate advantage over previously circulating lineages

For each of the sweeping lineages, that is those that rose from low to high frequency, we calculated the relative growth advantage compared to the background of all other lineages that were circulating at the same time (figure 1*c*). Because the ONS-CIS data were collected over periods during which the number of positive samples by lineage varied considerably depending on the phase of the epidemic curve, we developed a new method based on Gaussian processes. The code and details are available at https://github.com/thomasallanhouse/covid19-lineages, and the method can also be found in the electronic supplementary material. Importantly, because we compare a specific lineage to all other contemporary lineages, its growth rate advantage will depend on the composition of the background viral population and is not static in time. Hence a lineage which initially enjoys a growth rate advantage will eventually transition to having a disadvantage, as subsequent variants emerge and sweep through the population. Declining growth rate advantages after initial high values have also been noted in previous reports [23–27].

In line with previous findings [16,27–31] we found that all of the sweeping lineages, including B.1.177, had a significant growth rate advantage compared to all other co-circulating SARS-CoV-2 lineages during emergence, with the maximum per day advantage observed for each major lineage ranging from around 5% (a 14 day doubling time) for B.1.177, BA.2.75/Omicron, and XBB, to around 10% (7 day doubling time) for B.1.1.7/Alpha, B.1.617.2/Delta, BA.2/Omicron, BA.4/5/Omicron, and BQ.1, and around 33% (2 day doubling time) for BA.1/Omicron (figure 1*c*).

### (c) An increasing trend in Ct values from B.1.617.2/Delta onwards

It has previously been observed that when an epidemic is growing Ct values tend to be lower since a higher proportion of samples are taken from early infection when viral loads tend to be higher, and hence Ct values tend to be lower [32–35]. We observed similar trends in the median Ct values per week, with lower Ct values early on during new waves of infections, and higher Ct values later on in the waves (electronic supplementary material, figure S1). A drop in median Ct values during the B.1.1.7/Alpha wave is evident from the data, as was also observed in a detailed analysis of a subset of these data that accounted for epidemic growth [35].

In addition, taking Ct values from 1 June 2021 onwards, marking the beginning of the B.1.617.2/Delta wave through to the XBB wave, we observed an increasing trend in Ct values of 1.31 per year (linear regression, *p* << 0.001; electronic supplementary material, figure S1). A Ct increase of 3 equates to an approximately 10-fold decrease in viral burden [35], suggesting average viral burdens fell by approximately 6-fold during this period. This could be due to a number of factors, including increasing levels of population-level immunity, changes in the age distribution of who is infected and/or changing viral biology [35].

### (d) Alternating SGTF and non-SGTF

Curiously, the successive sweeps of distinct lineages observed in the UK, as also found in South Africa [16,31], have been characterized by the alternation of lineages that exhibit RT-PCR S-gene target failure (SGTF) caused by the Spike DH69/V70 deletion, with those that do not have SGTF (figure 1*d*; electronic supplementary material, figure S1). For sequenced samples with an assigned lineage, we classed those as having SGTF if, during RT-PCR testing, N and ORF1ab were successfully amplified but S was not, and non-SGTF samples as those where all three genes were amplified. Only samples where both N and ORF1ab were amplified were included in the SGTF analysis, and SGTF status was determined solely by the RT-PCR and not imputed from sequence data. Lineage was generally a good indicator of SGTF, with 98.84% (3416/3456) of B.1.1.7/Alpha, 99.82% (24511/24556) of BA.1/Omicron, 99.77% (3428/3464) of BA.4/Omicron and 99.53% (27106/27234) of BA.5/Omicron, including BQ.1, samples having SGTF; all four of these lineages have the DH69/V70 deletion. Other lineages, which generally lack the deletion, typically did not have SGTF; 0.48% (7/1450) of B.1.177 samples had SGTF, 0.16% (29/17766) of B.1.617.2/Delta, and 0.53% (226/42938) of BA.2/Omicron, including BA.2.75, and XBB. As previously reported [36], a clear exception was the B.1.258 lineage, of which around two-thirds of samples had both the DH69/V70 deletion and SGTF (48/71), and some B.1.617.2/Delta and BA.2 sequences also had the DH69/V70 deletion, and hence had SGTF (electronic supplementary material, figure S1).

The independent emergence of DH69/70 on different lineages, including B.1.258, B.1.1.7/Alpha, and BA.1/Omicron, is a prominent example of the convergence that has been observed repeatedly during the evolution of SARS-CoV-2 [37]. This pattern of alternating SGTF greatly facilitated the rapid quantification of the prevalence of different VOCs without the need for genome sequencing, enabling samples with high Ct to be included in epidemiological analyses, and avoiding delays associated with sequencing [16,24,38–42]; Intriguingly, the pattern of alternating SGTF and non-SGTF has continued until at least mid-March 2023, even though no new major lineages emerged, with BA.2 recombinant and sublineages that do not have SGTF (including XBB and BA.2.75) gradually outcompeting BA.5 and its sublineages (including BQ.1) which all have SGTF. As of mid-March 2023, XBB was the dominant lineage circulating in the UK.

However, SGTF or non-SGTF has not always been a reliable indicator of the lineage of a sample. This can be due to: (i) co-circulation of lineages with the same SGTF pattern such as B.1.358 and B.1.1.7/Alpha; BA.4 and BA.5; and BA.2.75 and XBB; (ii) lineages with sub-lineages that have different SGTF patterns, such as B.1.258; and (iii) a small proportion of samples being assigned the opposite to expected SGTF pattern—this will have a disproportionate impact when the prevalence of the lineage of interest is low while the prevalence of another lineage with the opposite SGTF pattern is high. This highlights the need for caution when using SGTF as a marker for lineage, particularly when prevalences are low, and the necessity of sequencing in obtaining a full understanding of the genetic landscape at any particular time.

### (e) Diversity increases within lineages through time, but fluctuates when measured across all lineages

With the exception of B.1.617.2/Delta, within-lineage diversity was generally low when each major lineage first appeared in the ONS-CIS, and gradually increased through time before replacement by a new variant (figure 1*e*). This initial low diversity is a consequence of their relatively recent emergence before first detection in the ONS-CIS data. On the other hand, B.1.617.2/Delta had high initial diversity (figure 1*e*), reflecting its repeated introduction from an already diverse source population in India [43].

In contrast, when we consider overall genetic diversity, we see transient increases, peaking when two or more major lineages are at relatively high frequencies (figure 1*e*), but then declining as single lineages dominate the population. This is unsurprising given the large number of mutations distinguishing the different major lineages, which will push up diversity when two or more lineages are relatively frequent. Nonetheless, it is striking that from the Autumn of 2020 to the Spring of 2023 we did not see a trend of increasing levels of global diversity despite nearly two and a half years of evolution during this time. The most sustained period of gradually increasing diversity was during the second half of 2022 as multiple sublineages of BA.2 and BA.5 co-circulated over a prolongued period of time, but this was then followed by a decline as the BA.2 recombinant lineage XBB began to dominate.

### (f) Divergence increases slower within than among lineages

**Figure 2.**
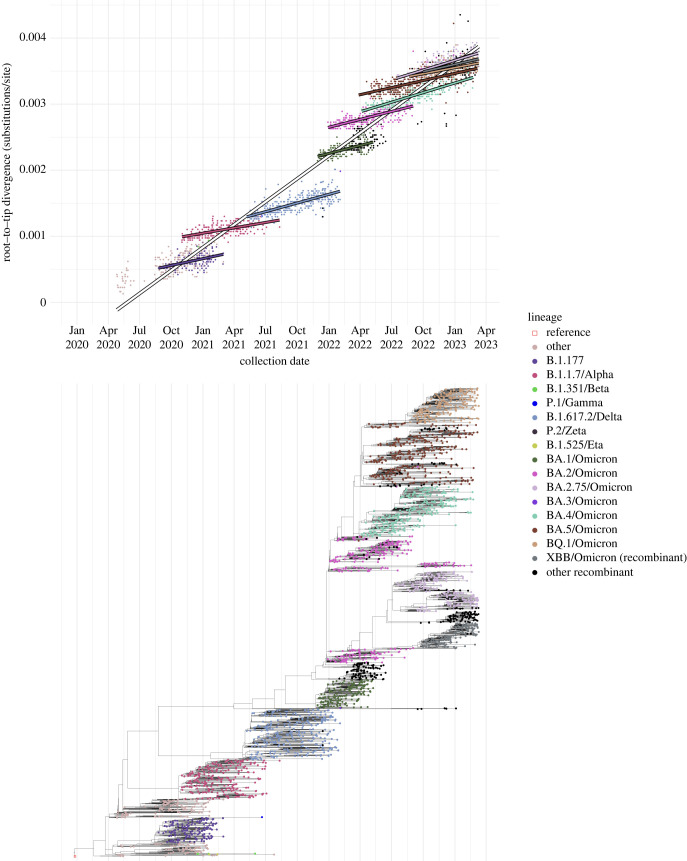
Dated phylogeny and root-to-tip distance of ONS-CIS sequences. A maximum likelihood phylogeny of 3000 ONS sequences with over 95% genome coverage was generated using IQ-TREE (electronic supplementary material, figure S3). The samples were chosen using weighted random sampling, ensuring the major lineages and the rarely samples VOCs were as evenly distributed through time as possible Top. Root to tip distance for samples from the maximum likelihood phylogeny. BA.2 sequences (excluding BA.2.75) collected before and after 12 September 2022 were considered separately; the later sequences are all from the BA.2.3.20 lineage or its descendents. Bottom. Time tree generated from the maximum likelihood phylogeny using TreeTime [45].

Phylogenies can be difficult to generate for large alignments, although a fast approximate maximum likelihood method has been developed specifically for SARS-CoV-2 [44]. These large phylogenies can also be subject to biases if lineages or epochs are unevenly sampled, and difficult to visualize. To overcome some of these issues, we subsampled 3000 high-coverage (>95%) consensus sequences from the ONS-CIS data using weighted random sampling, so that the VOCs, and the sweeping and partially sweeping lineages, were as similarly represented and as evenly distributed through time as possible. The sequential sweeps of the major lineages are readily observable on the resultant time-scaled phylogeny, with each of the major lineages representing a distinct clade or sub-clade (figure 2, electronic supplementary material, figure S2). The sampling methodology meant that lineages that were rarely sampled in the UK, such as B.1.351/Beta, B.1.525/Eta, P.1/Gamma, and BA.3/Omicron, were represented in the phylogeny. Apart from B.1.617.2/Delta, the major UK lineages have times of most recent common ancestor (tMRCAs) close to the time of first sampling in the ONS-CIS, indicating the recent emergence of these lineages when first sampled.

As expected, divergence from the root of the phylogeny increased gradually through time, both within-lineages and across all lineages (figure 2), demonstrating the presence of a strong molecular clock. The estimated overall mutation rate was 1.39 × 10^−3^ substitutions per site per year by simple linear regression. It has been noted previously that although divergence within the B.1.1.7/Alpha lineage increased at a similar rate to previously circulating lineages, it had accumulated a disproportionate number of lineage-defining mutations at the time of emergence [46], and that this pattern is also observed for other VOC lineages [47,48]. We can readily see this pattern using the subsampled ONS-sequences, and we formally investigate it using a linear regression of root-to-tip divergence versus calendar time, with lineage used as an interaction term (electronic supplementary material, table S1). B.1.177, used as the reference category, evolved at an estimated 4.06 × 10^−4^ substitutions per site per year, and there was no evidence that the rate of evolution differed for most other lineages (exceptions being BA.2.75 and BA.4, which were slightly faster). Substitution rates in the ‘Other’ category, which represents a diverse collection of early sequences and minor lineages, and whose estimate could be seen as representing between-lineage rate, was much faster (1.29 × 10^−3^ subs site^−1^ year^−1^). For this analysis, we split the BA.2 samples (excluding BA.2.75) into those collected prior to 12th September 2022 and those after. The later sampled lineages were contemporary to the BA.2.75 wave, and were members of the BA.2 sublineage BA.2.3.20, which harbours a large number of mutations compared to its predecessors, and are observed at a time when all other BA.2 lineages had effectively gone extinct.

The almost one-to-one concordance that we see between the emergence of lineages divergent compared to their predecessors, their growth rate advantage, and the resulting epidemic waves provides a clear narrative of epidemiological dynamics driven by saltational evolutionary events. Since new VOCs and other major lineages are characterized by nonsynonymous mutations [48], it has been hypothesized that they arose during long-term chronic infections with the virus subject to strong immune selection [49–51].

## 3. Discussion

Surveillance studies are valuable tools for tracking the emergence and spread of an infectious disease. Using sequenced samples collected as part of the ONS-CIS, we have demonstrated the utility of large-scale surveillance studies to identify key epidemiological and evolutionary features of the UK epidemic. Since participating households are chosen to be representative of the UK population, and enrolled individuals are periodically tested for SARS-CoV-2 infection regardless of symptoms, the ONS-CIS gives a picture of SARS-CoV-2 prevalence in the UK that is not subject to the biases arising from focussing on symptomatic individuals or other groups [6–8].

The sequential sweeps of different major lineages in the UK observed during the first three years of the UK epidemic resulted in a pattern of relatively steady within-lineage evolution and gradual increases in within-lineage diversity, followed by step-increases in the number of substitutions as each new major lineage emerged. This in turn produced faster estimates of overall rates of evolution when measured across all lineages, and fluctuating levels of genetic diversity. Whether this pattern will be an ongoing feature of SARS-CoV-2 evolution remains to be seen. However, it is noticeable that even though more recent major lineages have been descendants of previously circulating lineages (BA.2.75 and BA.2.3.20 are both descendents of BA.2, and BQ.1 is descended from BA.5), or recombinants, the large number of mutations these lineages have acquired compared to their predecessors is evident. If the hypothesis that these major lineages and other VOCs arose or partially arose from chronically-infected individuals is correct [46,50], it will be difficult to predict what the next sweeping lineage will look like, as it could potentially be a descendant of Omicron or of one of the earlier circulating lineages, including the more pathogenic B.1.617.2/Delta. This again emphasizes the need for effective genomic surveillance at a global scale.

A key observation from our study is that the growth rate advantage for a sweeping lineage will not be static in time. Instead, the relative growth rate of a variant will be determined by a number of factors, including its intrinsic transmissibility and the other viral lineages circulating at the time, but also immune escape and levels of population immunity to all extant variants [51]. It is interesting to note, for example, that during the Delta and Omicron epochs in our study Ct values gradually increased (viral burdens gradually decreased), and that higher Cts have previously been associated with lower transmissibility [52]. As a result, growth rate advantages may differ by both regions and calendar time, and there should be no *a priori* expectation that variants that have previously disappeared due to competition could not re-emerge, potentially seeded from chronically infected individuals, once the immunological background has changed.

It is tempting to argue that the alternating high prevalences of non-SGTF (B.1.177, B.617.2/Delta, BA.2) and SGTF lineages (B.1.1.7/Alpha, BA.1, BA.4/5), caused by the absence and presence of the DH69/V70 deletion, is a consequence of the changes in the immunological background. DH69/V70 causes conformational changes in the NTD loop and this in turn may compensate for, or have other epistatic interactions with, immune escape mutations [53]. The gradual increase in the proportion of BA.2 sequences that had DH69/V70 when BA.2 as a whole was in decline, and when BA.4 and BA.5 were beginning to sweep through the population, supports this idea, as does the resurgence of non-SGTF BA.2 recombinant and sub-lineages in the UK population towards the end of 2022, coinciding with a fall in BA.5 lineages. However, without a clear mechanism causing the switching, this remains a hypothesis only, and it is hard to predict whether the pattern will continue into the future.

A crucial drawback to genomic surveillance is the delay between sample collection and subsequent sequencing (in the ONS-CIS this was between two and three weeks) and the need for high viral load samples to produce adequate sequence data. This in turn could impact the success of any interventions. The earliest signals that both B.1.1.7/Alpha and BA.1/Omicron [31] had a growth rate advantage were serendipitously inferred from the increasing incidence of SGTF during RT-PCR testing, and conversely increasing non-SGTF was observed when B.1.617/Delta and BA.2 were emerging. However, the simultaneous rise in BA.4 and BA.5, both with SGTF, required sequencing to distinguish between them, and similarly the BA.2 recombinant and sublineages that subsequently overtook BA.4 and BA.5 cannot be distinguished in this way. The introduction of qPCR-based genotyping for specific lineages into diagnostic pipelines has the potential to speed-up detection of known variants [41], but the lead time required to manufacture them means they cannot be relied upon to characterize emerging variants fast enough to contain them. Moreover, substantial genome sequencing efforts will always be required to detect variants that have not previously been identified as of concern, to monitor the ongoing specificity of rapid genotyping in the face of ongoing evolution, and to better characterize the evolution and spread of the virus.

Although only a fraction of the COG-UK sequences were composed of samples collected as part of the ONS-CIS, we were able to use ONS-CIS sequenced samples to monitor the emergence, spread and evolution of the major lineages and sublineages sweeping through the UK population. Moving forwards, the implementation of genomic surveillance globally should be considered a key development goal, enabling the early detection of worrisome and/or rapidly growing lineages wherever they emerge. While community surveillence at the scale of the ONS-CIS is unlikely to be feasible in most regions of the world, the survey is an important benchmark, and provides data and lessons to be learnt for when designing surveillance studies in other countries. Incorporating the detection and sequencing of other pathogens into the same community surveillance frameworks will only act to enhance the positive public health and scientific outcomes from these studies while maximizing value for money.

## 4. Methods

### (a) Office for National Statistics Coronavirus (COVID-19) Infection Survey

The ONS-CIS is a UK household-based surveillance study whose participant households are chosen to provide a representative sample of the population. For a full description of the sampling design see [8], but in brief, swabs were taken from individuals aged two years and older, living in private households, from 26 April 2020 to 13 March 2023. These households were selected randomly from address lists and previous ONS surveys to provide a representative sample of the population. Participants could provide consent for optional follow-up sampling weekly for the first five weeks, and monthly thereafter.

This work contains statistical data from ONS which is Crown Copyright. The use of the ONS statistical data in this work does not imply the endorsement of the ONS in relation to the interpretation or analysis of the statistical data. This work uses research datasets which may not exactly reproduce National Statistics aggregates.

### (b) Sequencing

A selection of RT-PCR samples were sequenced each week, with some additional retrospective sequencing of stored samples. From December 2020 onwards, the ambition was to sequence all positive samples with Ct ≤ 30. Most samples were sequenced on Illumina Novaseq, but with a small number using Oxford Nanopore GridION or MINION. One of two protocols were used: either the ARTIC amplicon protocol [54] with consensus FASTA sequence files generated using the ARTIC Nextflow processing pipeline [54], or veSeq, an RNASeq protocol based on a quantitative targeted enrichment strategy [55,56] with consensus sequences produced using *shiver* [57].

For veSeq we have previously shown that viral load is positively correlated with the number of mapped reads, and that Ct is negatively correlated with the log10 number of mapped reads [32,35,55]. Where there was duplicate sequencing, either of the same sample or of multiple samples taken from an individual at the same time, only the sequence with the highest coverage was kept. Only sequences with >50% genome coverage were included in this analysis, and of these >125 000 sequences, approximately 2% had a sample Ct > 30. The proportion of swabs RT-PCR positive, RT-PCR positive and with Ct ≤ 30, and RT-PCR positive, Ct ≤ 30 and sequenced with >50% genome coverage is shown in electronic supplementary material, figure S1.

### (c) Lineage calling

Lineages using the Pango nomenclature [19] were determined using the Pangolin software [20]. Reported lineages include any sub-lineages, with the exception of BA.2, which did not include BA.2.75 and sublineages of BA.2.75, and BA.5 which did not include BQ.1 and sublineages of BQ.1. When comparing SGTF with lineage, we excluded samples where the lineage resolved to A, B, B.1 or B.1.1 since sequences can be given these Pango lineages if an insufficient number of loci have coverage at lineage defining sites. The COG-UK ID, collection dates, Pango lineage, and major lineage or rare VOC lineage, for all sequences included in this study can be downloaded from doi:10.5061/dryad.hx3ffbgm2, as well as the consensus sequences, labelled by the COG-UK ID and sampling date. The sequences have also been deposited in the European Nucleotide Archive (ENA) at EMBL-EBI as part of the COG-UK consortium, which has accession number PRJEB37886 (https://www.ebi.ac.uk/ena/browser/view/PRJEB37886).

### (d) Lineage growth rates and doubling times

For each of the major lineages, sublineages and recombinant lineages observed at high frequency, B.1.177, B.1.1.7/Alpha, B.1.617.2/Delta, the Omicron variants BA.1, BA.2 and BA.4 and BA.5, BA.2.75, BQ.1 and XBB, we calculated their relative growth rate advantage compared to all other lineages. BA.4 and BA.5 were considered together because of their concordant dynamics. We used a combination of Gaussian process regression and classification [58] together with bootstrapping as detailed fully at https://github.com/thomasallanhouse/covid19-lineages.

Our aim in analysing the growth rates of lineages is to determine their relative growth rate compared to other circulating lineages. The instantaneous value of this relative rate for a lineage is defined as

4.1 $$\delta r(t)=\;\frac{d}{dt}\log\left(\frac{\mu_{1}(t)}{\mu_{2}(t)}\right),$$

where $\mu_{1}(t)$ and $\mu_{2}(t)$ are estimates of the prevalence at time *t* for lineage of interest and all other lineages respectively. We show in the electronic supplementary material why this is the natural definition.

Since there is a time derivative in this expression, we cannot use raw daily prevalence counts and instead should use estimates that can be differentiated—in this case we use Gaussian processes as described by Rasmussen and Williams [58] and implemented in *scikit-learn* [59].

There are two ways to make estimates of equation (4.1), with performance depending on the number of samples associated with a lineage. For smaller datasets, we can use Gaussian process classification to provide an estimate of the proportion of all samples that are the lineage in question, π(t), and substitute the relation

4.2 $$\frac{\mu_{1}(t)}{\mu_{2}(t)}=\;\frac{\pi (t)}{1-\pi (t)},$$

into equation (4.1). For larger datasets, we can use Gaussian process regression to provide estimates $s_{1}$ and $s_{2}$ of $\log(\mu_{1}(t))\;$and $\log(\mu_{2}(t))$ respectively and then write

4.3 $$\delta r(t)=\;\frac{ds_{1}}{dt}-\frac{ds_{2}}{dt}.$$

Further details are given in the electronic supplementary material, Methods.

### (e) Nucleotide genetic diversity

Nucleotide genetic diversity was calculated using the π statistic, since this has been shown to be the least sensitive to differences in the number of sequences used in the analysis [60]. Mean pairwise genetic diversity across the genome is given by:

4.4 $$\pi =\frac{1}{L}\overset{L}{\underset{l=1}{\sum }}D_{l},$$

where *L* represents the length of the genome, and $D_{l}$ the pairwise genetic diversity at locus *l*. This is calculated as:

4.5 $$D_{l}=\frac{2}{N(N-1)}\underset{i\neq j}{\sum }n_{i}n_{j},$$

where $n_{i}$ represents the number of alleles *i* observed at that locus, and *N* the number of samples with a consensus base call. Within-lineage genetic diversity was similarly calculated as above, but limiting only to the major lineages.

### (f) Phylogenetics

For the phylogenetic analysis, 3000 consensus sequences with at least 95% coverage were chosen using weighted random sampling, with each sample of major lineage (or rare VOC lineage) *i* collected in week *j* given a weight *1/x_ij_*, where *x_ij_* is the number of sequences of lineage *i* collected during week *j*. The major lineages and rare VOC lineages including B.1.177, all of the identified VOCs (with BA.1 - BA.5 each given their own weighting), BA.2.75, BQ.1 and XBB. All other well-resolved lineages were placed in the category ‘Other’. The sub-sampled sequences are indicated in the table deposited at https://github.com/katrinalythgoe/ONSLineages. All were pairwise aligned to the Wuhan-Hu-1 reference strain using MAFFT [61] and then combined to generate a single alignment of 29 903 base pairs.

The alignment of 3000 sequences, combined with Wuhan-Hu-1, were used for phylogenetic reconstruction using IQ-TREE version 1.6.12 [62]. The substitution model used was GTR + F + R4 with four FreeRate rate categories, and the resulting tree was rooted using Wuhan-1 and then fit to the calendar using TreeTime version 0.8.2 [45]. Tips representing the reference strain and sequences judged by TreeTime as outliers on the root-to-tip divergence plot were pruned from the phylogeny and excluded from further analysis. Visualization used ggtree [63].

A linear regression analysis of root-to-tip divergence as a function of time since 1 January 2020 (in decimal years) and lineage was conducted. B.1.177 was used as the baseline category for lineage (electronic supplementary material, table S1). This included an interaction term for the two dependent variables. For this analysis, the BA.2 lineage was split into two (one for sequences sampled before 12 September 2022 and one for those after) and any lineages with less than ten examples among the 3000 were combined with the ‘Other’ category.

## Data Availability

The sequences are publicly available via the COG-UK project on the ENA, (https://www.ebi.ac.uk/ena/browser/view/PRJEB37886). The consensus sequences are also available as a FASTA file (https://doi.org/10.5061/dryad.hx3ffbgm2) [64], as is a table (‘SampleInfo.csv’) with the COG-UK ids, enabling them to be identified and download. The table also gives their collection dates, pangolin designation, major lineage, and whether they were included in the phylogenetic analysis. The weekly aggregated ONS-CIS testing data (‘TestingInfo.csv’) are also available here. The full description and code for the growth rate analysis is on T.H.'s github (https://github.com/thomasallanhouse/covid19-lineages). We have permission from the ONS to make these data publicly available. The data are provided in electronic supplementary material [65].

## References

[1] WHO. 2023 WHO global genomic surveillance strategy for pathogens with pandemic and epidemic potential 2022–2032. Accessed 2 June 2023. See https://www.who.int/initiatives/genomic-surveillance-strategy.

[2] UKHSA. 2023 Genome sequence prevalence and growth rate update: 24 May 2023. Accessed 2 June 2023. See https://www.gov.uk/government/publications/sars-cov-2-genome-sequence-prevalence-and-growth-rate/sars-cov-2-genome-sequence-prevalence-and-growth-rate-update-24-may-2023.

[3] Harvey WT, Carabelli AM, Jackson B, Gupta RK, Thomson EC, Harrison EM, Ludden C, Reeve R, Rambaut A. 2021 SARS-CoV-2 variants, spike mutations and immune escape. Nat. Rev. Microbiol. **19**, 409-424. (10.1038/s41579-021-00573-0) 34075212 PMC8167834

[4] Carabelli AM et al. 2023 SARS-CoV-2 Variant biology: immune escape, transmission and fitness. Nat. Rev. Microbiol. **21**, 162-177. (10.1038/s41579-022-00841-7) 36653446 PMC9847462

[5] The COVID-19 Genomics UK (COG-UK) consortium. 2020 An integrated national scale SARS-CoV-2 genomic surveillance network. Lancet Microbe. **1**, E99-E100. (10.1016/S2666-5247(20)30054-9) 32835336 PMC7266609

[6] Franceschi VB et al. 2021 Population-based prevalence surveys during the Covid-19 pandemic: a systematic review. Rev. Med. Virol. **31**, e2200. (10.1002/rmv.2200) 34260777 PMC7883186

[7] Kraemer MU, Cummings DA, Funk S, Reiner RC, Faria NR, Pybus OG, Cauchemez S. 2019 Reconstruction and prediction of viral disease epidemics. Epidemiol. Infect. **147**, e34. (10.1017/S0950268818002881) PMC6398585 30394230

[8] Pouwels KB et al. 2021 Community prevalence of SARS-CoV-2 in England from April to November, 2020: results from the ONS Coronavirus Infection Survey. Lancet. Public Health **6**, e30-e38. (10.1016/S2468-2667(20)30282-6) 33308423 PMC7786000

[9] Mohanan M, Malani A, Krishnan K, Acharya A. 2021 Prevalence of SARS-CoV-2 in Karnataka, India. JAMA **325**, 1001-1003. (10.1001/jama.2021.0332) 33538774 PMC7863009

[10] Wu SL et al. 2020 Substantial underestimation of SARS-CoV-2 infection in the United States. Nat. Commun. **11**, 1-10. (10.1038/s41467-020-18272-4) 32908126 PMC7481226

[11] Munnink O, Bas B, Worp N, Nieuwenhuijse DF, Sikkema RS, Haagmans B, Fouchier RAM, Koopmans M. 2021 The next phase of SARS-CoV-2 surveillance: real-time molecular epidemiology. Nat. Med. **27**, 1518-1524. (10.1038/s41591-021-01472-w) 34504335

[12] Sah P, Fitzpatrick MC, Zimmer CF, Abdollahi E, Juden-Kelly L, Moghadas SM, Singer BH, Galvani AP. 2021 Asymptomatic SARS-CoV-2 infection: a systematic review and meta-analysis. Proc. Natl Acad. Sci. USA **118**, e2109229118. (10.1073/pnas.2109229118) 34376550 PMC8403749

[13] UKHSA. 2021 Surge testing for new coronavirus (COVID-19) variants. See https://www.gov.uk/guidance/surge-testing-for-new-coronavirus-covid-19-variants.

[14] Elliot P et al. 2023 Design and implementation of a national SARS-CoV-2 monitoring program in England: REACT-1 study. Am. J. Public Health **113**, 545-554. (10.2105/AJPH.2023.307230) 36893367 PMC10088956

[15] Office for National Statistics. 2021 Coronavirus (COVID-19) Infection Survey: quality and methodology information (QMI). See https://www.ons.gov.uk/peoplepopulationandcommunity/healthandsocialcare/conditionsanddiseases/methodologies/coronaviruscovid19infectionsurveyqmi.

[16] Tegally H et al. 2022 Emergence of SARS-CoV-2 Omicron lineages BA.4 and BA.5 in South Africa. Nat. Med. **28**, 1-6. (10.1038/s41591-022-01911-2) 35760080 PMC9499863

[17] Subissi L et al. 2022 An early warning system for emerging SARS-CoV-2 variants. Nat. Med. **28**, 1110-1115. (10.1038/s41591-022-01836-w) 35637337 PMC11346314

[18] WHO. 2023 Tracking SARS-CoV-2 variants. Accessed 5 April 2023. See https://www.who.int/activities/tracking-SARS-CoV-2-variants.

[19] Rambaut A, Holmes EC, O'Toole Á, Hill V, McCrone JT, Ruis C, du Plessis L, Pybus OG. 2020 A dynamic nomenclature proposal for SARS-CoV-2 lineages to assist genomic epidemiology. Nature Microbiology **5**, 1403-1407. (10.1038/s41564-020-0770-5) PMC7610519 32669681

[20] O'Toole Á et al. 2021 Assignment of epidemiological lineages in an emerging pandemic using the Pangolin tool. Virus Evolution **7**, veab064. (10.1093/ve/veab064) 34527285 PMC8344591

[21] Office for National Statistics. 2021 Coronavirus (COVID-19) infection survey: technical data. See https://www.ons.gov.uk/peoplepopulationandcommunity/healthandsocialcare/conditionsanddiseases/datasets/covid19infectionsurveytechnicaldata/2021.

[22] Vihta KD et al. 2021 Symptoms and severe acute respiratory syndrome coronavirus 2 (SARS-CoV-2) positivity in the general population in the United Kingdom. Clin. Infect. Dis. **71**, e329-e337. (10.1093/cid/ciab945) PMC8767848 34748629

[23] Kraemer MUG et al. 2021 Spatiotemporal invasion dynamics of SARS-CoV-2 lineage B.1.1.7 emergence. Science **373**, 889-895. (10.1126/science.abj0113) 34301854 PMC9269003

[24] Volz E et al. 2021 Assessing transmissibility of SARS-CoV-2 lineage B.1.1.7 in England. Nature **593**, 266-269. (10.1038/s41586-021-03470-x) 33767447

[25] Davies NG et al. 2021 Estimated transmissibility and impact of SARS-CoV-2 lineage B.1.1.7 in England. Science **372**, eabg3055. (10.1126/science.abg3055) 33658326 PMC8128288

[26] Jones TC et al. 2021 Estimating infectiousness throughout SARS-CoV-2 infection course. Science. **372**, eabg3055. (10.1126/science.abi5273) 34035154 PMC9267347

[27] Vöhringer HS et al. 2021 Genomic reconstruction of the SARS-CoV-2 epidemic in England. Nature **600**, 506-511. (10.1038/s41586-021-04069-y) 34649268 PMC8674138

[28] Lemey P et al. 2021 Untangling introductions and persistence in COVID-19 resurgence in Europe. Nature **595**, 713-717. (10.1038/s41586-021-03754-2) 34192736 PMC8324533

[29] Hodcroft EB et al. 2021 Spread of a SARS-CoV-2 variant through Europe in the summer of 2020. Nature **595**, 707-712. (10.1038/s41586-021-03677-y) 34098568

[30] Sonabend R et al. 2021 Non-pharmaceutical interventions, vaccination, and the SARS-CoV-2 Delta variant in England: a mathematical modelling study. Lancet. **398**, 1825-1835. (10.1016/S0140-6736(21)02276-5) 34717829 PMC8550916

[31] Viana R et al. 2021 Rapid epidemic expansion of the SARS-CoV-2 Omicron variant in Southern Africa. Nature **603**, 679-686. (10.1038/s41586-022-04411-y) PMC8942855 35042229

[32] Golubchik T et al. 2021 Early analysis of a potential link between viral load and the N501Y mutation in the SARS-COV-2 spike protein. medRxiv 20249080. (10.1101/2021.01.12.20249080)

[33] Hay JA, Kennedy-Shaffer L, Kanjilal S, Lennon NJ, Gabriel SB, Lipstich M, Mina MJ. 2021 Estimating epidemiologic dynamics from cross-sectional viral load distributions. Science **373**, eabh0635. (10.1126/science.abh0635) 34083451 PMC8527857

[34] Lin Y et al. 2022 Incorporating temporal distribution of population-level viral load enables real-time estimation of COVID-19 transmission. Nat. Commun. **13**, 1155. (10.1038/s41467-022-28812-9) 35241662 PMC8894407

[35] Fryer HR et al. 2023 Viral burden is associated with age, vaccination, and viral variant in a population-representative study of SARS-CoV-2 that accounts for time-since-infection related sampling bias. PLoS Pathog. **19**, e1011461. (10.1371/journal.ppat.1011461) 37578971 PMC10449197

[36] Thomson EC et al. 2021 Circulating SARS-CoV-2 spike N439K variants maintain fitness while evading antibody-mediated immunity. Cell **184**, 1171-1187. (10.1016/j.cell.2021.01.037) 33621484 PMC7843029

[37] McCarthy KR, Rennick LJ, Nambulli S, Robinson-McCarthy LR, Bain WG, Haidar G, Paul Duprex W. 2021 Recurrent deletions in the SARS-CoV-2 spike glycoprotein drive antibody escape. Science **371**, 1139-1142. (10.1126/science.abf6950) 33536258 PMC7971772

[38] Kidd M et al. 2021 S-Variant SARS-CoV-2 Lineage B1.1.7 is associated with significantly higher viral load in samples tested by TaqPath polymerase chain reaction. J. Infect. Dis. **223**, 1666-1670. (10.1093/infdis/jiab082) 33580259 PMC7928763

[39] Walker AS et al. 2021 Increased infections, but not viral burden, with a new SARS-CoV-2 variant. medRxiv 21249721. (10.1101/2021.01.13.21249721)

[40] Davies NG, Jarvis CI, John Edmunds W, Jewell NP, Diaz-Ordaz K, Keogh RH. 2021 Increased mortality in community-tested cases of SARS-CoV-2 lineage B.1.1.7. Nature **593**, 270-274. (10.1101/2021.02.01.21250959) 33723411 PMC9170116

[41] Vogels CBF et al. 2021 Multiplex qPCR discriminates variants of concern to enhance global surveillance of SARS-CoV-2. PLoS Biol. **19**, e3001236. (10.1371/journal.pbio.3001236) 33961632 PMC8133773

[42] Office for National Statistics. 2021 Coronavirus (COVID-19) Infection Survey, UK: 16 July 2021. See https://www.ons.gov.uk/peoplepopulationandcommunity/healthandsocialcare/conditionsanddiseases/bulletins/coronaviruscovid19infectionsurveypilot/16july2021.

[43] Public Health England. 2021 SARS-CoV-2 variants of concern and variants under investigation in England: technical briefing 10. See https://assets.publishing.service.gov.uk/government/uploads/system/uploads/attachment_data/file/984274/Variants_of_Concern_VOC_Technical_Briefing_10_England.pdf.

[44] Turakhia Y, Thornlow B, Hinrichs AS, De Maio N, Gozashti L, Lanfear R, Haussler D, Corbett-Detig R. 2021 Ultrafast sample placement on existing trees (UShER) enables real-time phylogenetics for the SARS-CoV-2 pandemic. Nat. Genet. **53**, 809-816. (10.1038/s41588-021-00862-7) 33972780 PMC9248294

[45] Sagulenko P, Puller V, Neher RA. 2018 TreeTime: maximum-likelihood phylodynamic analysis. Virus Evolution **4**, vex042. (10.1093/ve/vex042) 29340210 PMC5758920

[46] Hill V et al. 2022 The origins and molecular evolution of SARS-CoV-2 lineage B.1.1.7 in the UK. Virus Evolution **8**, veac080. (10.1093/ve/veac080) 36533153 PMC9752794

[47] Tay JH, Porter AF, Wirth W, Duchene S. 2022 The emergence of SARS-CoV-2 variants of concern is driven by acceleration of the substitution rate. Mol. Biol. Evol. **39**, msac013. (10.1093/molbev/msac013) 35038741 PMC8807201

[48] Neher RA. 2022 Contributions of adaptation and purifying selection to SARS-CoV-2 Evolution. Virus Evolution **8**, veac113. (10.1093/ve/veac113) 37593203 PMC10431346

[49] Rambaut AN, Loman, Pybus O, Barclay W, Barrett J, Carabelli A, Connor T. 2020 Preliminary genomic characterisation of an emergent SARS-CoV-2 lineage in the UK defined by a novel set of spike mutations. 18 December. See https://virological.org/t/preliminary-genomic-characterisation-of-an-emergent-sars-cov-2-lineage-in-the-uk-defined-by-a-novel-set-of-spike-mutations/563.

[50] Ghafari M et al. 2023 High number of SARS-CoV-2 persistent infections uncovered through genetic analysis of samples from a large community-based surveillance study. medRxiv. (10.1101/2023.01.29.23285160)

[51] Markov PV, Ghafari M, Beer M, Lythgoe K, Simmonds P, Stilianakis NI, Katzourakis A. 2023 The evolution of SARS-CoV-2. Nature Reviews: Microbiology **21**, 361-379. (10.1038/s41579-023-00878-2) 37020110

[52] Lee LYW et al. 2022 Severe acute respiratory syndrome coronavirus 2 (SARS-CoV-2) infectivity by viral load, S gene variants and demographic factors, and the utility of lateral flow devices to prevent transmission. Clinical Infectious Diseases **74**, 407-415. (10.1093/cid/ciab421) 33972994 PMC8136027

[53] Meng B et al. 2021 Recurrent emergence of SARS-CoV-2 spike deletion H69/V70 and its role in the Alpha variant B.1.1.7. Cell Reports **35**. (10.1016/j.celrep.2021.109292) PMC8185188 34166617

[54] COG-UK. 2020 nCoV-2019 sequencing protocol V.1. See https://www.protocols.io/view/ncov-2019-sequencing-protocol-bp2l6n26rgqe/v1.

[55] Lythgoe KA et al. 2021 SARS-CoV-2 within-host diversity and transmission. Science **372**, eabg0821. (10.1126/science.abg0821) 33688063 PMC8128293

[56] Bonsall D et al. 2020 A comprehensive genomics solution for HIV surveillance and clinical monitoring in low-income settings. J. Clin. Microbiol. **58**, 10-128. (10.1128/JCM.00382-20) PMC7512176 32669382

[57] Wymant C et al. 2018 Easy and accurate reconstruction of whole HIV genomes from short-read sequence data with Shiver. Virus Evolution **4**, vey007. (10.1093/ve/vey007) 29876136 PMC5961307

[58] Rasmussen CE, Williams CKI. 2005 Gaussian processes for machine learning. Cambridge, MA: MIT Press.

[59] Pedregosa F et al. 2011 Scikit-learn: Machine learning in Python. J. Machine Learning Res. **12**, 2825-2830. (https://scikit-learn.org/stable/)

[60] Zhao L, Illingworth CJR. 2019 Measurements of intrahost viral diversity require an unbiased diversity metric. Virus Evolution **5**, vey041. (10.1093/ve/vey041) 30723551 PMC6354029

[61] Katoh K, Standley DM. 2013 MAFFT multiple sequence alignment software version 7: improvements in performance and usability. Mol. Biol. Evol. **30**, 772-780. (10.1093/molbev/mst010) 23329690 PMC3603318

[62] Minh BQ, Schmidt HA, Chernomor O, Schrempf D, Woodhams MD, von Haeseler A, Lanfear R, Teeling E. 2020 IQ-TREE 2: new models and efficient methods for phylogenetic inference in the genomic era. Mol. Biol. Evol. **37**, 1530-1534. (10.1093/molbev/msaa015) 32011700 PMC7182206

[63] Yu G, Smith DK, Zhu H, Guan Y, Lam TT. 2017 Ggtree: an R package for visualization and annotation of phylogenetic trees with their covariates and other associated data. Methods Ecol. Evol. **8**, 28-36. (10.1111/2041-210X.12628)

[64] Lythgoe KA et al. 2023 Data from: Lineage replacement and evolution captured by three years of the United Kingdom COVID infection survey. Dryad Digital Repository. (10.5061/dryad.hx3ffbgm2) PMC10581763 37848057

[65] Lythgoe KA et al. 2023 Lineage replacement and evolution captured by 3 years of the United Kingdom Coronavirus (COVID-19) Infection Survey. Figshare. (10.6084/m9.figshare.c.6837585) PMC10581763 37848057

